# ^13^C-NMR-Based Metabolomic Profiling of Typical Asian Soy Sauces

**DOI:** 10.3390/molecules21091168

**Published:** 2016-09-02

**Authors:** Ghulam Mustafa Kamal, Bin Yuan, Abdullah Ijaz Hussain, Jie Wang, Bin Jiang, Xu Zhang, Maili Liu

**Affiliations:** 1 Key Laboratory of Magnetic Resonance in Biological Systems, State Key Laboratory of Magnetic Resonance and Atomic and Molecular Physics, Centre for Magnetic Resonance, Wuhan Institute of Physics and Mathematics, Chinese Academy of Sciences, Wuhan 430071, China; kamal_ss@hotmail.com (G.M.K.); yuankanxue@163.com (B.Y.); jie.wang@wipm.ac.cn (J.W.); jbin@wipm.ac.cn (B.J.); 2 University of Chinese Academy of Sciences, Beijing 100049, China; 3 Institute of Chemistry, Government College University Faisalabad, Faisalabad 38000, Pakistan; abdullahijaz@gmail.com

**Keywords:** soy sauce, NMR, metabolomics, PCA, OPLS-DA, metabolites, fermentation

## Abstract

It has been a strong consumer interest to choose high quality food products with clear information about their origin and composition. In the present study, a total of 22 Asian soy sauce samples have been analyzed in terms of ^13^C-NMR spectroscopy. Spectral data were analyzed by multivariate statistical methods in order to find out the important metabolites causing the discrimination among typical soy sauces from different Asian regions. It was found that significantly higher concentrations of glutamate in Chinese red cooking (CR) soy sauce may be the result of the manual addition of monosodium glutamate (MSG) in the final soy sauce product. Whereas lower concentrations of amino acids, like leucine, isoleucine and valine, observed in CR indicate the different fermentation period used in production of CR soy sauce, on the other hand, the concentration of some fermentation cycle metabolites, such as acetate and sucrose, can be divided into two groups. The concentrations of these fermentation cycle metabolites were lower in CR and Singapore Kikkoman (SK), whereas much higher in Japanese shoyu (JS) and Taiwan (China) light (TL), which depict the influence of climatic conditions. Therefore, the results of our study directly indicate the influences of traditional ways of fermentation, climatic conditions and the selection of raw materials and can be helpful for consumers to choose their desired soy sauce products, as well as for researchers in further authentication studies about soy sauce.

## 1. Introduction

Naturally and historically, food products are land based, and food consumption habits are generally shaped by the socio-cultural factors and the available natural resources of a specific region. In the last few years, it has been a growing interest among consumers based both in developed and developing countries to purchase food products that are deeply rooted in popular cultures or simply come from a specific reputed place of manufacture. A Swiss evaluation has revealed that the origin of food is a criterion for 82% of consumers [1]. For small and medium-sized companies, this new trend could imply new opportunities to differentiate product in the market and secure price premiums and/or increased sales [2,3].

Soy sauce is a traditional oriental food having a salty taste and distinct fragrance, which is used as a condiment or seasoning sauce worldwide. Its production consists of solid-state fermentation of plant materials and so-called brine fermentation [4]. The use of soy sauce dates back 2000 years. However, the use of soybean as food was introduced to the eastern half of north China in the 11th century BC [5]. In China, soy sauce is produced by the fermentation of steamed soybean and raw wheat flour with *Aspergillus oryzae* (44 h of koji fermentation), which yields moromi in 20%–25% brine (4–6 months of moromi fermentation). Ripened moromi is then pressed to yield a liquid called soy sauce [6,7]. During the course of the long fermentation period, a characteristics taste and aroma is imparted to soy sauce. However, soy sauce products from different geographic regions possess unique sensory and textural properties caused by the different raw materials and manufacturing processes used. Thus, the characteristics of soy sauces differ according to their geographic region of origin and fermentation procedures [8]. Red cooking soy sauce, a popular soy sauce in China made by the extra addition of rock sugar rendering a deep savory flavor, is made especially for stir fried or red cooking dishes; whereas, super light soy sauce, another popular type of naturally-brewed soy sauce common in Mainland China, as well as Taiwan (China) and Singapore, is usually considered as an all-purpose soy sauce. Naturally-brewed low salt light soy sauce from Taiwan (China) was selected for our study. Kikkoman, a Japanese-based soy sauce manufacturer, has manufacturing plants in many countries and produces a variety of soy sauces according to the varying cultural needs. Kikkoman soy sauce samples under our study were imported from Singapore and represented low salt naturally-brewed Kikkoman soy sauce. Shoyu, a traditional naturally-brewed soy sauce famous in Japan, is produced by following a centuries-old fermentation procedure involving a koji (*Aspergillus* *oryzae*) converting the hard to digest soy and wheat proteins to easily-absorbed amino acids. Various types of soy sauces available in global market have their own specific appeal for the consumers.

With the increased number of available types of soy sauces offering a range of different flavorings, additives and sweeteners, the curiosity of consumers about the choice and composition of the product has also been increased nowadays. Several reports are available in the literature regarding the studies on bioactive amines, isoflavones, amino acids, flavoring and taste compounds, phytochemicals and toxic compounds in soy sauce [9,10,11,12]. A few reports are available for the studies of geographic origin or differentiation among types of soy sauce. Aishima [13,14] used near infrared (NIR) spectra and chemometrics pattern recognition for the classification of Japanese soy sauces by geographic region. However, all of these techniques are specific to a typical class of compounds and require a time-consuming sample preparation.

Metabolomics has presently been applied as a promising method to improve the understanding of food materials, like composition differentiation, geographical variations and processing diversity, that may lead to food quality control and authentications [15,16]. NMR coupled with multivariate statistical analysis is one of the major tools used for the metabolomic studies. NMR spectroscopy being robust, non-selective and nondestructive provides maximum information about the sample without requiring any complex sample preparation procedures. The combination of NMR fingerprinting with chemometric methods provides an original approach to study the profile of a food product in relation to its geographical origin [17,18].

In the present study, ^13^C-NMR spectroscopy combined with advanced chemometric methods was used to provide the consumer with easy, rapid and robust information for the differentiation among some of the most popular Asian soy sauces based on manufacturing processes (fermentation methods, raw materials, addition of preservatives, additives and flavorings).

## 2. Results and Discussion

### 2.1. ^13^C-NMR Spectroscopy

**Figure 1:**
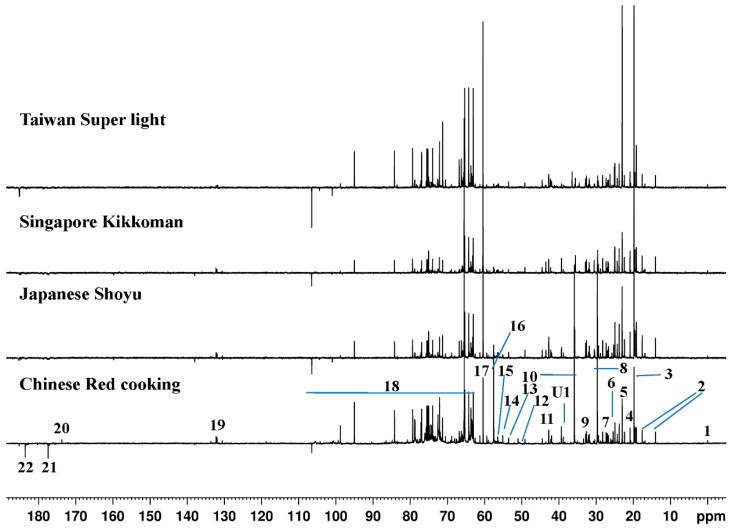
Representative ^13^C-NMR spectra of four types of Asian soy sauces viz. Chinese red cooking, Japanese shoyu, Singapore Kikkoman and Taiwan super light. Peaks: 1: trimethylsilyl propanoic acid (TSP); 2: isoleucine; 3: alanine; 4: valine; 5: threonine; 6: acetate; 7: leucine; 8: glutamate; 9: isoleucine; 10: leucine; u1: unknown; 11: leucine; 12: malonate; 13: betaine; 14: choline; 15: aspartate; 16: glutamate; 17: valine; 18: glucose and sucrose; 19: tyrosine; 20: phenylalanine; 21: lactate; 22: acetate.

Figure 1 shows the representative 1D ^13^C-NMR spectra of Chinese red cooking (CR), Japanese shoyu (JS), Singapore Kikkoman (SK) and Taiwan light (TL) soy sauces. Although no clear and apparent differences were observed in the overall pattern of the spectra, distinct differences in composition and concentrations of specific metabolites were observed among species. No serious overlaps were observed in ^13^C-NMR spectra, which are often observed in ^1^H-NMR spectra due to oligosaccharide overlaps. Therefore, the ^13^C-NMR spectroscopy was considered to be useful for the metabolomics of soy sauce. It has been shown previously that ^13^C-NMR spectroscopy would be useful in metabolomics by providing complementary component information, while potentially reducing the problems of overlap that are common in ^1^H-NMR spectroscopy [19,20]. However, using DEPT, the spin sensitivity of ^13^C is enhanced by polarization transfer via scalar coupling from ^1^H (I) to ^13^C (S). The transfer of polarization is highly dependent on one coupling constant of bonds (^1^*J*_SI_), spin system (IS_1_, IS_2_, IS_3_) and transfer delay (Δ). The sensitivity of ^13^C spin enhancement is different because of the different nature of protons attached to the carbons, and thus, ^1^*J*_SI_ values narrow down the use of polarization transfer for quantitative studies used for metabolomics [21]. At the same time, the long *T*_1_ relaxation time reduces signal intensities, which sometimes result in challenges in the quantification. However, for metabolomic purposes in which all samples are measured under the same conditions, such quantification is less necessary if the *T*_1_ times are maintained constant, because it reveals the overall pattern of response that can be interpreted [18,22]. This technique relies on transferring polarization from protons to the X-nucleus, except quaternary carbon. Since the proton magnetization is the original signals being transferred, the relaxation time of carbon, except quaternary carbons, is irrelevant. The repetition rate of the experiment is determined by proton *T*_1_. The signals of the quaternary carbon are less sensitive and have much longer *T*_1_ (^13^C) than other signals, so we only pay attention to the signals of the other carbons and set up the experiment with a faster repetition rate, like 2 s. In this condition, the quantification of most of the metabolites is reliable, except a few with quaternary carbons. Therefore, the data resulting from DEPTQ ^13^C-NMR spectra can be utilized for the calculation of the relative concentration (in terms of normalized peak areas) of the metabolites using the same representative signal for each metabolite from all spectra. The assignments of the metabolites have been carried out making use of 2D NMR (^1^H-^1^H TOCSY and ^1^H-^13^C HSQC), the online NMR spectral databases, like Spectral Database for Organic Compounds (SDBS), Madison Metabolomics Consortium Database (MMCD) and Biological Magnetic Resonance Databank (BMRB), and already published information [23]. A total of 22 metabolites’ signals were identified in the ^13^C-NMR spectra of all types of soy sauce with different ranges of concentration (Table 1). The variation in metabolite concentration might be linked to the different traditional ways of manufacturing, like the selection of raw materials, the fermentation procedure and the addition of different types of additives (preservatives, sweeteners and salts) and flavors.

### 2.2. Multivariate Statistical Analysis

**Figure 2:**
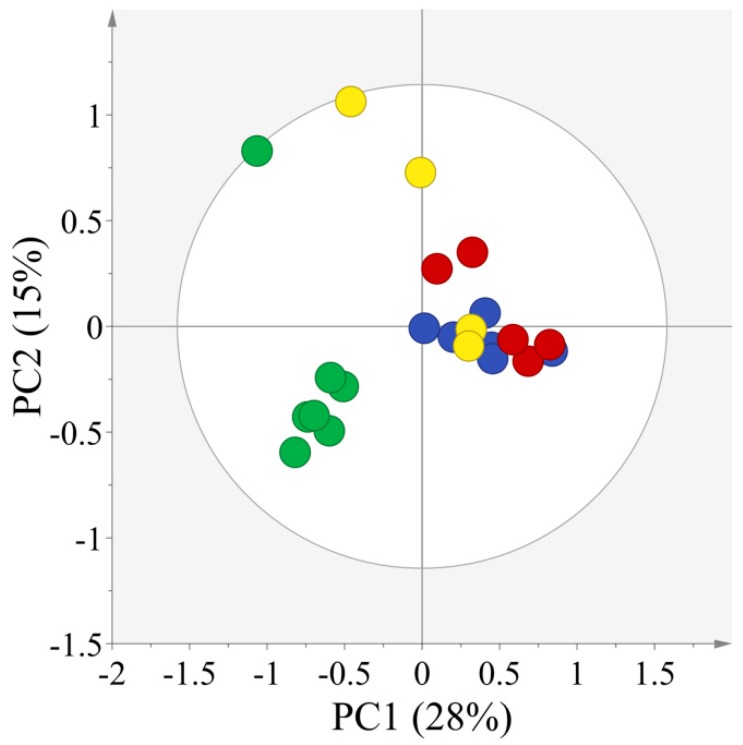
PCA score scatter plot derived from ^13^C-NMR spectra of all types of Asian soy sauces showing the separation of Chinese red cooking soy sauce (green circle) from those of Japanese shoyu (blue circle), Singapore Kikkoman (red circle) and Taiwan super light soy sauce (yellow circle).

In order to expose differences in the concentration of metabolites in soy sauce from different origins, the ^13^C-NMR spectra of 22 samples of soy sauce were then analyzed by PCA. PCA, which is an unsupervised classification method, requires no previous information of the dataset and yields a screening model to reduce the dimensionality of data, while saving most of the variance within it [24]. The PCA score scatter plot with R2X (54%) and Q2 (20%) is shown in Figure 2, which is derived from the ^13^C-NMR spectra of four typical Asian soy sauces namely; CR, TL JS and SK. R2X represents the goodness of fit, and Q2 is the predictability of the PCA model [25].

The PCA model showed a clear separation of CR soy sauce from JS, SK and TL soy sauce, indicating the uniqueness of red cooking soy sauce (Figure 2); while no clear separation was observed among TL, SK and JS soy sauces.

Therefore, the main differences were observed among CR and the other three types of soy sauce from Japan, Singapore and Taiwan (China). This was observed because the CR soy sauce is a subtype of super dark soy sauce, whereas JS, SK and TL basically belong to the super light category of soy sauce. The fermentation procedures, addition of additives, flavorings, etc., are generally different for light and dark types of soy sauce.

In order to exploit different metabolomic characteristics of the four types of soy sauce, pairwise OPLS-DA models were calculated for CR soy sauce with JS, SK and TL soy sauce because the main differences were found among CR and the other three types of soy sauce. The important variables responsible for discrimination among different types of soy sauce were captured by OPLS-DA loading line plots.

**Figure 3:**
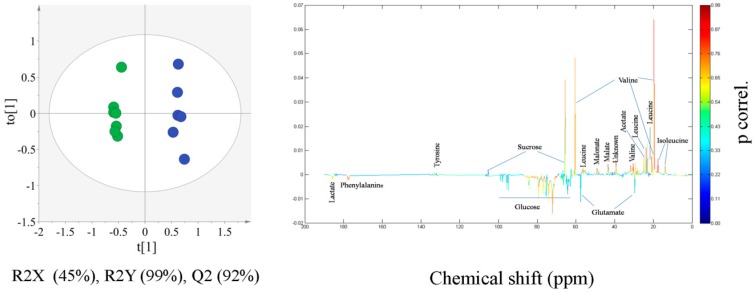
OPLS-DA score scatter plot derived from ^13^C-NMR spectra of Chinese red cooking (green circle) and Japanese shoyu (blue circle) soy sauce and the color-coded loading line plot generated from the OPLS-DA model representing the metabolites responsible for discrimination among Chinese red cooking and Japanese shoyu soy sauce.

OPLS-DA model calculated for comparison of CR and JS is shown in Figure 3. Figure 3 shows the separation of CR from JS soy sauce with statistical significance of R2X (Cum.) 47%, R2Y (Cum.) 99% and Q2 (Cum.) 92%. Variables responsible for separation in the OPLS-DA scatter plot were captured by the loading line plot shown in Figure 3. The loading line plot helps identify statistically and potentially biochemically significant variables, on the basis of both their contribution and reliability. The significant variables were identified according to the ^13^C-NMR assignment information from Figure 1. Metabolites corresponding to the significantly discriminating variables are isoleucine, leucine, valine, acetate, malonate, malate, glucose, lactate and phenylalanine. This suggested that compared with CR, JS contained significantly higher levels of isoleucine, leucine, valine, acetate, malonate and malate, whereas CR soy sauces were characterized by increased concentrations of glucose, phenylalanine and lactate.

**Figure 4:**
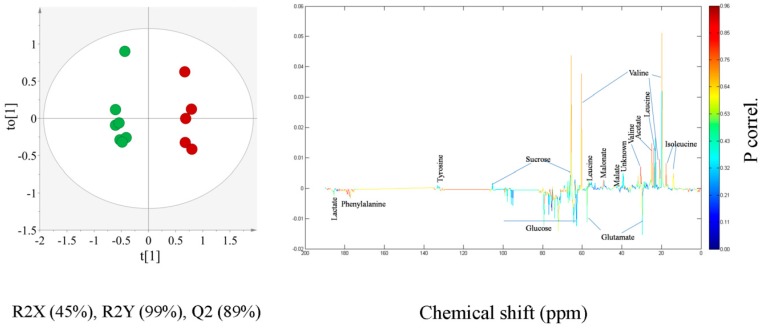
OPLS-DA score scatter plot derived from ^13^C-NMR spectra of Chinese red cooking (green circle) and Singapore Kikkoman (red circle) soy sauce and the color-coded loading line plot generated from the OPLS-DA model representing the metabolites responsible for discrimination among Chinese red cooking and Singapore Kikkoman soy sauce.

To clearly identify the underlying variables for the separation among CR and SK soy sauce, another OPLS-DA model was constructed (Figure 4) making use of data from CR and SK soy sauce samples. The OPLS-DA score plot (Figure 4) showed a clear separation among the two types with statistical significance of R2X (Cum.) 47%, R2Y (Cum.) 99% and Q2 (Cum.) 91%. The metabolites contributing significantly to this separation in the OPLS-DA score plot were captured by calculating a corresponding OPLS-DA line loading plot shown in Figure 4. From the loading line plot, it was evident that the concentrations of isoleucine, valine, acetate and malonate are significantly higher in SK, whereas CR soy sauce was characterized with a significantly increased level of phenylalanine.

**Figure 5:**
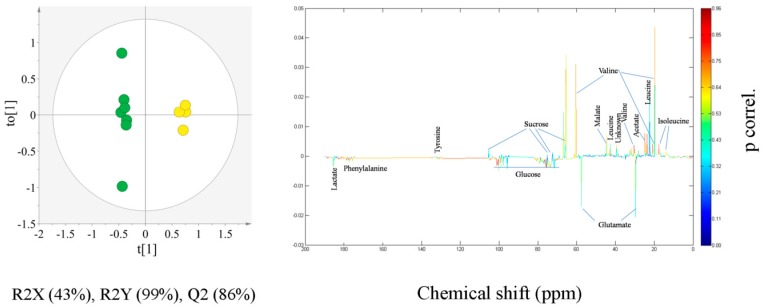
OPLS-DA score scatter plot derived from ^13^C-NMR spectra of Chinese red cooking (green circle) and Taiwan super light (yellow circle) soy sauce and the color-coded loading line plot generated from the OPLS-DA model representing the metabolites responsible for discrimination among Chinese red cooking and Taiwan super light soy sauce.

To observe the underlying differences among CR and TL soy sauce, a third OPLS-DA model was calculated using the ^13^C-NMR data from the two types of soy sauce (Figure 5). The OPLS-DA score plot (Figure 5) highlighted the statistically-significant differences among the CR and TL soy sauce with R2X (Cum.) 77%, R2Y (Cum.) 100% and Q2 95%. To find out the underlying variables responsible for this separation in the OPLS-DA score plot, a loading line plot was calculated (Figure 5). The OPLS-DA loading plot revealed significantly increased levels of isoleucine, valine, acetate and malate in TL, whereas significantly higher levels of glucose and phenylalanine were detected in CR soy sauce.

To further clarify the characterization of other metabolites, except glutamate, all two-class OPLS-DA models were conducted again by excluding all resonances from glutamate (data not shown). The results from all of the models were consistent for the variations of the other metabolites mentioned above, and the differences of CR from JS, SK and TL were still feasible. This confirms that most of the contribution towards CR is not only from the glutamate.

**Figure 6:**
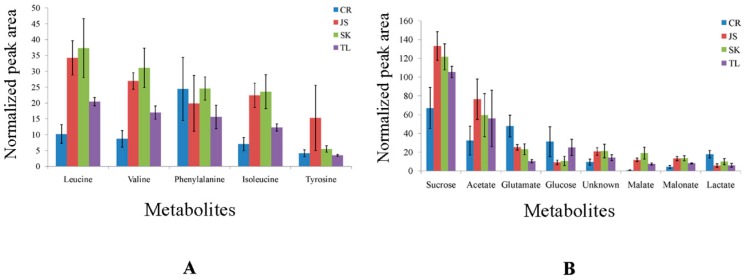
(**A**) Relative concentrations of discriminating amino acid metabolites (in terms of normalized peak areas) captured by OPLS-DA models derived from the ^13^C-NMR spectra of Chinese red cooking, Japanese shoyu, Singapore Kikkoman and Taiwan super light soy sauce; (**B**) Relative concentrations (in terms of normalized peak areas) of significantly-different metabolites (excluding amino acids) captured by OPLS-DA models derived from the ^13^C-NMR spectra of Chinese red cooking, Japanese shoyu, Singapore Kikkoman and Taiwan super light soy sauce.

To confirm and discuss the importance of the discriminating metabolites captured by OPLS-DA loading line plots, relative concentrations of the biomarkers (in terms of their relative normalized peak areas) were plotted in standard error bar graphs (Figure 6A,B). The distinctions among different types of Asian soy sauces can be explained on the basis of different processing techniques, like fermentation methods (traditional or acidic hydrolysis), raw materials from different origins and the addition of additives, preservative and taste developers.

Significantly different levels of amino acids, like leucine, isoleucine and valine, were observed in different types of soy sauce in the present study (Figure 6A). The differences in types and concentrations of amino acids may be linked to the difference in fermentation periods and the activity of the bacteria. Amino acids in soy sauce are produced by the microbial activity during the course of fermentation. The microorganisms break down the soy and wheat proteins into amino acids through proteolysis and peptidase activity and use them as a nitrogen source for their growth. The same was observed in significant consumption and synthesis of valine in grape wine fermentation [26]. The concentrations of most of the amino acids, like leucine, isoleucine and valine, were found lower in CR, indicating the shorter fermentation period, since the raw materials used in CR show not much difference with the others. There are certain soy sauce products that are not naturally brewed. These contain processed products produced by enzymes or other additives, such as chemicals, like acids, that break down proteins in order to speed up fermentation. Hydrolyzed products have their own demand in terms of taste, and because of this, the industry standards for soy sauce are recently under debate by the international Codex Alimentarius Commission. Though CR contained the lowest concentration of amino acids as compared to the other three types of soy sauce, however, the concentrations of the important amino acids, like isoleucine, phenylalanine and tyrosine, were almost comparable between CR and SK soy sauce. Similar trend was observed among JS and TL in terms of the concentrations of leucine, valine, phenylalanine and isoleucine.

A significantly higher concentration of phenylalanine was observed in CR soy sauce (Figure 6A). Phenylalanine is produced during fermentation and is categorized as a bitter amino acid. It imparts an intense umami taste along with glutamate [7]. Glutamate was also observed in significantly higher (almost two-fold) concentration in CR soy sauce samples as compared to JS, SK and TL soy sauce (Figure 6B). Thus, phenylalanine along with glutamate caused an intense umami taste in CR soy sauce. This was also confirmed by tasting the CR soy sauce samples. Glutamate is an important amino acid present both in raw and fermented soy sauce [27] and serves as a key intermediate in lactic acid bacteria (LAB) metabolism because of its utilization by most of the aminotransferases as the donor of amino groups. Glutamate is also produced by glutamate dehydrogenase during the course of fermentation, from 2-oxoglutarate, a tricarboxylic acid (TCA) metabolism intermediate.

Both the glutamic acid and glutamates are flavor enhancers and umami (savory) taste condiments found extensively in foods. Glutamate is an essential part of many fermented foods, like soy sauce, fermented beans, cheese, etc. Glutamate salts, such as monosodium glutamate (MSG), are a widely-used additive in Chinese soy sauce, which impart characteristic umami taste in combination with bitter phenylalanine; though MSG is not considered as a healthy additive if present in concentrations higher than the permitted level. A number of in vitro studies state that glutamate is a strong neurotoxin if present at higher concentrations and can destroy neurons by apoptosis [28]. Hence, it is important to disclose the reality of whether the MSG and glutamine-rich raw material, like wheat gluten instead of soybean, were used or the glutamate was produced by the fermentation process.

A higher concentration of tyrosine was found to be in JS as compared to CR, SK and TL soy sauce (Figure 6A). Though the difference was not significant, this may be important to explain the dynamics of fermentation processes. Tyrosine is often transformed to tyramine by microbial decarboxylation in fruits and vegetables during the fermentation process [29]. The lower levels of tyrosine in a fermented product provide evidence of the longer aging period of the soy sauce fermentation, indicating the conversion of tyrosine to tyramine. Tyrosine and its metabolites impact human mental health, adjusting to stress, blood pressure, skin color, ability to stand pain and metabolic rate [30,31].

Higher concentrations of acetate were detected in JS, SK and TL soy sauce (Figure 6B). Acetate generates during fermentation by osmotolerant LAB along with formate and causes inhibition in the growth of halophilic yeasts, such as *Saccharomyces* *rouxii* and *Torulopsis* *versatilis*, in Japanese soy sauce shoyu, and of *Shigella* [32,33,34]. Increased levels of acetate show the prolonged fermentation period during the production of JS and TL soy sauce.

A significantly higher concentration (almost two-fold) of lactate was observed in CR soy sauce as compared to JS, SK and TL soy sauce (Figure 6B). Increased levels of lactate suggest that halophilic or osmoprotolerant lactic acid bacteria were involved in the brine fermentation of soy sauce, and this indicates the traditional fermentation [35]. Lactate or lactic acid is the main organic acid and very important in determining the quality of soy sauce. It is produced naturally during fermentation or sometimes added manually by some manufacturers. Lactic acid gives a mild and balanced acidic flavor to soy sauce with some lingering effects. It gives a refined, rounded tartness, which is thought to be one of the important factors of a good soy sauce flavor.

CR soy sauce was characterized with higher levels of glucose, whereas, JS, TL and SK contained increased amounts of sucrose (Figure 6B). However, this difference in the concentration of glucose was non-significant. A lesser concentration of sugars in a fermentation product indicates the increased consumption of carbohydrates by microorganisms during the fermentation process [26]. Previous studies suggested oligosaccharide consumption by wine yeast in grape wines and a reduction in oligosaccharides concentration during the six months of alcoholic fermentation. Moreover, carbohydrates in soy sauce are helpful in the synthesis of immunoglobulin A (Ig A) in vivo and in vitro [36]. Similarly, *Tetragenococcus halophilus* is a halophilic lactic acid bacterium (LAB), which is active in the fermentation processes of soy sauce and possesses immunomodulatory activity in vitro [37,38]. In this way, soy sauce can serve as an important dietary source of improving host defenses, as halophilic or osmophilic bacteria degrade soybean and wheat starches into polysaccharides and oligosaccharides over a long period. A substantial amount of sugars is however required to be reserved in soy sauce in order to impart a peculiar taste, so the fermentation process should be properly optimized. Elevated levels of carbohydrates in this way can support the idea that the fermentation period of the product was shorter. However, some manufacturers add caramel to increase the viscosity and some sweeteners to improve the taste of the final soy sauce product. In addition to the microbial activities, the variations in glucose and sucrose concentrations among different kinds of Asian soy sauces can also be linked to the manual additions of caramel and some sweeteners by the manufacturers.

SK and TL soy sauce were characterized with significantly increased amounts of malonate as compared to CR soy sauce (Figure 6B). Malate was also observed in significantly higher concentrations in JS, SK and TL soy sauce as compared to CR soy sauce (Figure 6B). Malate is produced from fumarate by the action of fumarase enzyme through the TCA cycle in the fermentation process. It is a reversible reaction, and on oxidation catalyzed by malate dehydrogenase, malate converts to oxaloacetate [39]. Malonate is also produced in the TCA cycle and acts as a contender of succinate dehydrogenase without reacting with it. Different levels of organic acids and their esters in typical Asian soy sauces indicate the choices of different starter cultures, brining and aging strategies for fermentation. Decreased amounts of TCA cycle intermediates like malate and malonate also support the idea of a shorter fermentation period adopted for the production of CR soy sauce.

Except glutamate, lactate and glucose, there was a comparable trend in the change in concentration of some important fermentation metabolites (acetate, sucrose and malonate) among CR and SK. The same trend was observed among JS and TL. The similar trends in the change in concentration of most of the amino acids and of the other fermentation cycle intermediates suggests that there might be similarities in the manufacturing methods of CR and SK and similarly among those of JS and TL. Taiwan (China) and Japan have some climatic similarities (subtropical monsoon climate). Similarly, Singapore is located in the South China Sea. The red cooking soy sauces we bought are produced from manufactures based in south China, which are similar in climate and cultures to Singapore. This may be the reason for the similar trends found in the concentration of soy sauce products from these regions.

An unknown compound was also detected in higher concentration in JS, TL and SK soy sauce, whereas a low content of the unknown compound was detected in Chinese red cooking soy sauce. However, the differences in the concentration of the unknown compound among different types of Asian soy sauce were not significant.

## 3. Experimental Section

### 3.1. Chemicals

All chemical reagents were of analytical grade. Deuterium oxide (D_2_O) (99.9%), trimethylsilylpropanoic acid (TSP) sodium salt (99.9 atom %), Na_2_HPO_4_ and NaH_2_PO_4_ were purchased from Sigma (St. Louis, MO, USA).

### 3.2. Sample Collection and Preparation

Popular soy sauces from Chinese mainland (red cooking soy sauce, CR = 7 samples) and Taiwan (super light, TL = 4 samples) and imported from Japan (Japanese Shoyu, JS = 6 samples) and Singapore (Singapore Kikkoman, SK = 5 samples) were purchased from local Chinese market. The detailed information about the samples selected is given in Table 2. The pH of all of the samples was monitored and adjusted to 5.0 ± 0.05 by mixing 60 µL soy sauce with 480 µL phosphate buffer (0.05 M sodium phosphate, pH 5.0 ± 0.05). After that, 60 µL 2.5 mM trimethylsilylpropanoic acid (TSP) prepared in D_2_O were added, where D_2_O provided field frequency lock, while TSP served as the internal reference standard. The samples were then centrifuged at 13,000 rpm for 10 min. The supernatants were transferred to 5 mm NMR tubes for further analysis.

### 3.3. ^1^H- and ^13^C-NMR Analysis

^1^H and ^13^C NMR spectra were all acquired on a Bruker Avance 600MHz NMR spectrometer (Bruker Biospin GmbH, Rheinstetten, Germany) operating at the ^1^H and ^13^C frequency of 600.1699 and 150.912 MHz, respectively, at a temperature of 298 K using a TXICryo Probe. For the ^1^H-NMR spectra, the H_2_O signal was suppressed by the presaturation method, and the parameters for observation were as follows: number of collected data points, 32 K; spectral width; 9000 Hz; acquisition time, 1.8 s; repetition time, 1.00 s; number of scans, 128. To get ^13^C-NMR signals, a QDEPT135 pulse sequence was optimized and applied. Eight thousand transitions for each sample were collected in 32 K data points using a spectral width of 240 ppm with a relaxation delay of 2.00 s and an acquisition time of 0.45 s. Chemical shifts of all of the signals were calibrated to TSP (^1^H and ^13^C, σ 0.00). All of the ^1^H and ^13^C spectra were apodized using the exponential window function with a line broadening factor of 0.3 and 3.0 Hz prior to Fourier transformation (FT), respectively. Signal assignment for representative samples was facilitated by 2D ^1^H-^1^H total correlation spectroscopy (TOCSY), hetero-nuclear single quantum correlation spectroscopy (HSQC) and online spectral databases.

### 3.4. NMR Data Analysis

Chemical shift migrations present among different NMR spectra are one of the most disturbing factors keeping in view the multivariate data analysis [40]. The exploration of patterns in the spectra is generally disturbed due to this problem [41,42,43]. Instrumental factors, changes of the salt or specific ionic concentration, pH and temperature, are among the most important factors causing this shift. However, these impacts on the chemical shifts of different resonances are not the same. To counterfeit the alignment and phase problems, all spectra were first manually phased and baseline corrected by Topspin software 3.2 (Bruker Biospin) and reduced into 0.2-ppm spectral buckets and integrated. Data files were then re-aligned using an in-house-developed software package NMRSpecs (version 1.0, Wuhan Institute of Physics and Mathematics, Wuhan, China), which can be used free of cost for academic research purposes [44]. The regions without signals (0–10 ppm, 137–175 ppm and 190–230 ppm) were removed during the spectral alignment and bucketing. In order to compensate for the total concentration differences, the aligned and bucketed spectra were normalized to the total spectral area for each spectrum.

### 3.5. Multivariate Data Analysis

The spectral data resulting from alignment and bucketing were imported to SIMCA 14 (Umetrics, Umea, Sweden) for conducting the multivariate statistical analysis. The par scaling was applied during all multivariate analyses. PCA, an un-supervised pattern recognition analysis, was conducted at first to reveal the intrinsic variations in the dataset and to diagnose any possible outliers. Following the variations found in PCA, OPLS-DA models were then calculated to find out the discriminating metabolites among different types of soy sauces. The statistical quality of the model was defined by the total variance of the two components at a confidence level of 95%. The overall predictability of the model is shown by cumulative Q2 representing the fraction of the variation of the Y that can be predicted by the model, which was extracted according to the internal default cross-validation method of the SIMCA software. The method used to measure these variations is simple and robust, with a general applicability to data mining from metabolomic and other similar kinds of datasets.

### 3.6. Relative Concentration of Metabolites

The normalized peak areas of selected metabolite signals were compared by using the integrals of representative signals (the least overlapping ones) relative to that of internal reference TSP with a known concentration.

The peak areas of each metabolite relative to the area of TSP peak were plotted in a standard error bar graph to clearly explain the variation among different types of soy sauce in terms of relative concentration (Figure 6A,B).

## 4. Conclusions

In summary, the present study demonstrated that ^13^C-NMR-based metabolomics is a useful and potential tool for distinguishing different types of soy sauce manufactured through different methods and that it highly improves sample classification when combined with chemometrics. The study highlights that typical soy sauce types manufactured in different regions can easily and rapidly be discriminated by evaluating the major metabolites quantitatively using metabolomic profiling. In our findings, the significantly different concentrations of amino acids, like leucine, isoleucine and valine, and of other fermentation cycle intermediates, like lactate, acetate, malate and malonate, among CR and JS, SK and TL indicate different lengths or types of fermentation used for the production of CR as compared to the other three types of Asian soy sauces. Significantly increased concentration of glutamate in Chinese (Mainland) red cooking samples of soy sauce indicates the manual addition of monosodium glutamate to the final soy sauce product. Comparable trends in the variation in concentrations of fermentation cycle metabolites among CR, SK and JS, TL indicate the influences of traditional ways of fermentation and a climate of geographically-similar conditions. Our findings can be helpful for the consumers to choose their desired soy sauce products, as well as for researchers in further authentication studies about soy sauce.

## Figures and Tables

**Table 1:** Metabolites and their ^13^C chemical shifts identified by the 600-MHz NMR spectrometer.

Compound	^13^C-NMR Chemical Shift	Group
Isoleucine	13.98, 17.54 *	–C6H_3_, –C5H_3_
Alanine	19.74	–C3H3
Valine	20.81 *, 60.38	–C4H_3_, –C2H
Threonine	22.33	–C4H_3_
Acetate/Acetic acid	22.97 *, 185	–CH_3_, –CO
Glutamate	29.67, 57.53 *	–C5H_3_, –C3H
Leucine	26.72, 42.68 *	–C4H, C3H_3_
Unknown	39.23–39.35 *	—
Malate	44 *	C4H_3_
Malonate	49.10 *	C2H_3_
Betaine	56.22	N(CH_3_)_3_
Choline	56.43	N(CH_3_)_3_
Aspartate	56.70	–C3H
Glucose	63–105 (94.99 *)	—
Sucrose	63–105 (65.48 *)	—
Tyrosine	131–132 *	–C3H
Phenylalanine	176–177 *	–C=O
Lactate	184 *	–C=O

The chemical shifts were determined at pH 5 ± 0.05 and expressed as relative values to those of TSP at 0 ppm. * Signals used for the comparison of significantly-differing metabolite concentrations among different types of soy sauce.

**Table 2:** Information about the samples.

Soy Sauce Type	Raw Materials	Fermentation Process
Chinese red cooking	Steamed soybean and wheat, caramel color and or molasses, spice extracts, MSG, sodium benzoate.	Prolonged fermentation of steamed soybeans and wheat with *A. oryzae*.
Japanese Shoyu (Tamari)	Less wheat and more soybean, alcohol as preservative.	Stronger alcoholic fermentation with yeast.
Singapore Kikkoman	Low salt, water, soybean, less wheat, rice, alcohol.	Brine fermentation with yeast or *Aspergillus* and lactic acid bacteria.
Taiwan (China) light	Black soybeans, wheat, water, less salt, sodium benzoate.	Japanese-style fermentation.
